# Molecular mechanisms of gastric epithelial cell adhesion and injection of CagA by *Helicobacter pylori*

**DOI:** 10.1186/1478-811X-9-28

**Published:** 2011-11-01

**Authors:** Steffen Backert, Marguerite Clyne, Nicole Tegtmeyer

**Affiliations:** 1 University College Dublin, School of Biomolecular and Biomedical Sciences, Science Center West, Belfield Campus, Dublin-4, Ireland; 2 University College Dublin, School of Medicine and Medical Science, Health Science Center, Belfield Campus, Dublin-4, Ireland

**Keywords:** *Helicobacter pylori*, adherence, adhesin, integrin, receptor, signalling, type IV secretion

## Abstract

*Helicobacter pylori *is a highly successful pathogen uniquely adapted to colonize humans. Gastric infections with this bacterium can induce pathology ranging from chronic gastritis and peptic ulcers to gastric cancer. More virulent *H. pylori *isolates harbour numerous well-known adhesins (BabA/B, SabA, AlpA/B, OipA and HopZ) and the *cag *(cytotoxin-associated genes) pathogenicity island encoding a type IV secretion system (T4SS). The adhesins establish tight bacterial contact with host target cells and the T4SS represents a needle-like pilus device for the delivery of effector proteins into host target cells such as CagA. BabA and SabA bind to blood group antigen and sialylated proteins respectively, and a series of T4SS components including CagI, CagL, CagY and CagA have been shown to target the integrin β_1 _receptor followed by injection of CagA across the host cell membrane. The interaction of CagA with membrane-anchored phosphatidylserine may also play a role in the delivery process. While substantial progress has been made in our current understanding of many of the above factors, the host cell receptors for OipA, HopZ and AlpA/B during infection are still unknown. Here we review the recent progress in characterizing the interactions of the various adhesins and structural T4SS proteins with host cell factors. The contribution of these interactions to *H. pylori *colonization and pathogenesis is discussed.

## Introduction

*H. pylori *colonises the stomach of about half of the human world population, which is associated with chronic, often asymptomatic gastritis in all infected individuals. Depending on various criteria, more severe gastric diseases including peptic ulcer disease can occur in up to 10-15% of infected persons [1-3]. *H. pylori *infections are commonly diagnosed with a strong inflammatory response, but the bacteria evolved numerous mechanisms during evolution to avoid recognition and clearance by the host defence machineries, and if not treated with antibiotics, they can persist for life. *H. pylori*-induced gastritis is the strongest singular risk factor for developing cancers of the stomach; however, only a small proportion of infected individuals develop malignancy such as mucosa-associated lymphoid tissue (MALT) lymphoma and even gastric adenocarcinoma [1-3]. Gastric adenocarcinoma constitutes the second leading cause of cancer-associated death worldwide, and about 700,000 people die from this malignancy annually [3]. The clinical outcome of infection with *H. pylori *is dependent on a very complex scenario of host-pathogen crosstalk. Disease progression is determined by various factors including the genetic predisposition of the host, the bacterial genotype and environmental parameters [1-3]. The cellular and molecular mechanisms developed by *H. pylori *to undermine host defence strategies have been under intense investigation worldwide.

Clinical *H. pylori *strains are highly diverse both in their genetic information and potential to induce pathogenicity. Myriads of bacterial factors have been reported to influence the pathogenesis of *H. pylori *infections. There are two classical virulence determinants expressed by *H. pylori*, the CagA protein encoded by the cytotoxin-associated genes pathogenicity island (*cag*PAI) and the vacuolating cytotoxin (VacA). Secreted VacA can trigger various responses including pore formation in the host cell membrane, modification of endo-lysosomal trafficking, cellular vacuolation, immune cell inhibition and apoptosis. VacA's activities are highlighted in several review articles [1-4] and will not be discussed here. In the mid nineties, the *cag*PAI was entirely sequenced from various *H. pylori *strains and found to represent a 40-kb DNA insertion element in the chromosome, which is flanked by 31-bp direct repeats and carrying up to 32 genes [5,6]. Large scientific interest concentrates on the CagA protein which is present in more virulent isolates, but is typically absent in less virulent *H. pylori *strains. Thus, CagA serves as a virulence marker for the *cag*PAI [7,8]. Work in the last ten years has shown that the *cag*PAI encodes type-IV secretion system (T4SS) which injects CagA into target cells where it interferes with multiple host cell signaling cascades [9,10]. Other well-described pathogenicity-associated mechanisms include flagella-driven bacterial motility, urease-mediated neutralization of pH, HtrA-mediated cleavage of E-cadherin, modification of host cell cholesterol, shedding of outer-membrane vesicles and peptidoglycan-dependent immune responses [1-3,11-13]. In addition, *H. pylori *carries several classical surface adhesins permitting tight adherence of the bacteria to gastric epithelial cells. Here we review the various molecular adhesion strategies of *H. pylori *to gastric epithelial target cells which facilitate bacterial binding. We also discuss the structure and function of the T4SS, and how it makes contact with host cell surface factors to inject the CagA effector protein.

## Role of the classical *H. pylori *adhesins

**Figure 1:**
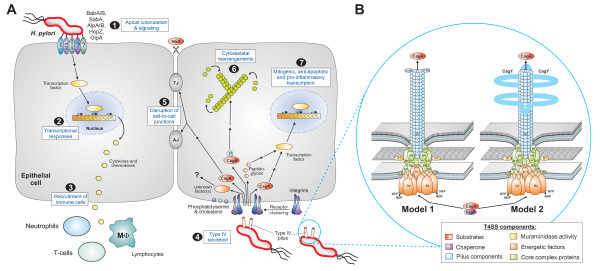
***H. pylori *interactions with epithelial cells highlighting the roles of adhesins and the type-IV secretion system (T4SS) encoded by *cag*PAI**. (A) (1) *H. pylori *adhesins mediate apical binding to known and unknown receptors on gastric epithelium and probably also direct signal transduction as indicated. (2) Upregulation of transcription factors such as NF-κB leads to production of pro-inflammatory cytokines and chemokines. (3) Secretion of mediators at basolateral surfaces attracts immune cells to the site of infection. (4) Upon host cell contact, *H. pylori *assembles T4SS pili at their surface enabling delivery of molecules, CagA and peptidoglycan, from bacterial cytoplasm into host cells. *cag*PAI proteins (CagA, CagI, CagL and CagY) interact with integrin receptors. Interactions with phosphatidylserine (PS) and cholesterol in lipid rafts are also involved in T4SS processes. T4SS and CagA are involved in numerous cellular effects including disruption of cell-to-cell junctions (5), cytoskeletal rearrangements (6) and nuclear signalling (7). (**B**) Two models for the assembled T4SS machinery in *H. pylori *are proposed. Model-1 assumes VirB1-11 proteins, the coupling factor VirD4 and accessory factors such as CagF (a proposed chaperone of CagA) assemble in a similar fashion to that proposed for *A. tumefaciens *T4SS [10]. Model-2 assumes that the T4SS requires the same VirB/D proteins as model-1 with two major differences. The T4SS pilus surface is covered with CagY (VirB10) molecules and VirB5 is excluded [50]. *H. pylori *VirB10 is a very large protein (~250 kDa) carrying two transmembrane domains to form a hairpin-loop structure in the pilus as depicted [64]. Immunogold labelling of the loop region in CagY indicated that this is exposed to the extracellular space and is transported to the pilus surface by an unknown mechanism [64]. Abbreviations: AJ (adherens junction); HtrA (High temperature requirement A protease); Leb (Lewis B antigens); MΦ (macrophage); NTP (nucleotide triphosphate); NDP (nucleotide diphosphate); P (phosphate group); SDL (sialyl-dimeric-Lewis × glycosphingolipid); TJ (tight junction).

Intensive research in recent years has demonstrated that *H. pylori *encodes a broad set of various adhesion factors, for some of which the corresponding host cell receptor(s) have been identified (Table 1). The *H. pylori *genomes from various strains contain more than 30 genes which encode outer membrane proteins (OMPs) that have been divided into Hop (*Helicobacter *outer membrane porins) and Hor (hop-related) subgroups. The Hop family of proteins includes several well described adhesins of *H. pylori *such as BabA, SabA, AlpA/B, HopZ and OipA. However, among clinical strains of *H. pylori *considerable diversity in the expression of OMPs exists. This is thought to reflect a selective pressure for bacterial adhesion which may differ both across and within infected individuals over time. It has been shown that some of the classical adhesion molecules discussed below act in conjunction with factors from the *cag*PAI in order to highjack several host cell processes including altered transcription, cytoskeletal rearrangements, opening of cell-to-cell junctions, onset of inflammation and others as summarized in a simplified model (Figure 1A).

**Table 1:** Characteristics of *cag*PAI-independent and *cag*PAI-dependent host cell adhesion factors^a^

Bacterial factor	Reported host receptor	Cell systems used	*H. pylori *strains used	Applied methods	References
BabA	Fucosylated blood group antigens Leb, and H1	Human gastric tissue sections	P466, CCUG17875, A5, M019, 26695	RIA, Receptor displacement asssay, Scatchard analysis,	[14]
	Leb, H1, A, ALeb, and BLeb	-	P466, CCUG17875, J99	Overlay binding studies with radiolabelled glycoconjugates	[15]

BabB	Unknown	-	-	-	-

SabA	Sialylated dimeric Lewis x, Sialyl Lea antigen	Human and monkey gastric tissue sections	CCUG17875, J99, 26695, SM165, WU12	RIA and Scatchard analysis	[26]
	Laminin	-	J99	Glycoconjugate array binding studies	[30]

OipA	Unknown	AGS, KATO-III	B128, G27	Bacterial adherence assays	[37]

AlpA/B	Laminin	-	26695, SS1	Flow cytometry, Biacore binding studies, surface adherence assay	[33]

HopZ	Unknown	AGS	ATCC43504, 342, 326/01	AGS cell adhesion assays	[39]

CagA	β1 integrin	GE11 vs. GE11β1	P12	Y2H, PD with magnetic beads, FACS, Biacore binding studies	[82]
	Phosphatidylserine	AGS	NCTC11637	Binding studies, CF, AB blocking, IF	[83]

CagI	β1 integrin	-	-	Y2H, PD with magnetic beads, FACS	[82]

CagL	β1 integrin	AGS, GD25 vs. GD25β1, HeLa, mouse fibroblasts	P1, P12	Biacore binding studies, Peptide competition, cell adhesion assays, AB blocking, CF	[61,81]

CagY	β1 integrin	GE11 vs. GE11β1	P12	Y2H, PD with magnetic beads, FACS	[82]

^a ^Abbreviations: AB (antibody); CF (cellular fractionation); FACS (Fluorescence-activated cell sorting); IF (co-localization by immunofluorescence); PD (pull-down experiments); RIA (Radioimmuno assay); Y2H (yeast two hybrid screen).

## BabA

The OMP member BabA was the first *H. pylori *adhesin discovered. BabA mediates binding of the bacteria to Lewis B antigens, Leb [14] and related terminal fucose residues found on blood group O (H antigen), A and B antigens [15] that are expressed on the gastric mucosa. Multiple chromosomal loci and alleles for BabA have been reported to exist and Leb binding activity was shown to be facilitated by the BabA2 allele [14]. However, more recent work suggests that BabA1 alleles occur only very rarely and are difficult to detect [16] Substantial amino acid polymorphisms exist among BabA proteins expressed by different strains [17]. Diversity also appears with respect to the binding of strains to Leb and BabA expression, and binding affinity for A, B and O antigens correlates with blood group antigen expression of the host [15,18]. A closely related gene *babB*, encodes for a translation product which has significant N- and C-terminal similarity to BabA. *BabA *and *babB *are nearly identical in their 5' and 3' regions but there is sequence divergence in their mid region [19] indicating that the central variable regions likely encode unique functions. Many Leb non-binding strains express silent *babA *gene sequences which may become activated by recombination into the *babB *locus forming chimeric *babB/A *genes [20]. BabA expression *in vivo*, however, appears to be highly dynamic. Experimental infection of Rhesus Macaques, mice and Mongolian gerbils resulted in loss of BabA expression and Leb binding [21,22]. Post-infection strains isolated from gerbils contained a BabA2 protein that was modified by six amino acids from the strain used for inoculation. Complementation experiments confirmed these six amino acid residues are critical for binding to fucosylated blood group antigens [22]. In a recent study, experimental infection of Mongolian gerbils resulted in complete absence of expression of BabA at six months post-infection. Loss of BabA expression was attributable to nucleotide changes within the *babA *gene that resulted in a truncated BabA translation product [23]. BabA-mediated adherence of *H. pylori *to Leb on the surface of epithelial cells has been shown *in vitro*, using Leb transfected MDCK cells, and *in vivo*, using infection of Mongolian gerbils, to augment *cag*PAI-dependent *H. pylori *pathogenicity by triggering the production of proinflammatory cytokines and precancer-related factors [24] (Figure 1A). Thus, the expression of the BabA adhesin seems tightly connected to the onset of T4SS-related host cell responses *in vivo*. The presence of *babA *is associated with *cagA *and *vacA *s1 alleles, and strains that possess all three of these genes incur the highest risk of gastric cancer development [25].

## SabA

Expression of sialyl-dimeric-Lewis × glycosphingolipid is upregulated upon infection with *H. pylori *and inflammation. This molecule also acts as a receptor for the pathogen and binding is mediated through the bacterial OMP member, SabA [26]. No binding to gangliosides was obtained with a SabA-negative mutant strain using a thin layer chromatography overlay assay [27]. Infection of the gastric epithelial cancer cell line MKN45 with *H. pylori *upregulated expression of the gene encoding β 3 GlcNAcT5, a GlcNAc transferase essential for the biosynthesis of Lewis antigens. Overexpression of this gene in both the MKN45 and AGS gastric adenocarcinoma cell lines lead to expression of the SabA ligand, sialyl Lex, suggesting that *H. pylori *can modulate receptor expression [28]. SabA has been identified as a haemagglutinin that binds to sialylated structures found on the surface of red blood cells and there is a good correlation among strains between sialic acid dependent haemagglutination and sialyl Lex binding [29]. Like observations with BabA, a high level of polymorphism was reported in sialyl binding properties among clinical *H. pylori *isolates, which suggests that SabA adapts to its host depending on the mucosal sialylation pattern of the infected individual [29]. SabA has also been shown to mediate binding of *H. pylori *to sialylated moieties on the extracellular matrix protein laminin [30].

## AlpA/B

Two strongly homologous genes termed *alpA *and *alpB *were also characterized and shown to encode for integral OMPs. Adhesion experiments indicated that they are also involved in adherence of *H. pylori *to human gastric tissue biopsies [31]. OMP expression profiling of 200 strains from Germany revealed that virtually all clinical isolates produced the AlpA and AlpB proteins in contrast to many other OMPs that were produced at very variable rates [32]. Recently both AlpA and AlpB proteins have been shown to bind to mouse laminin *in vitro *and plasmid-borne *alpA *conferred laminin-binding ability on *E. coli *[33]. No other binding partners or receptors for AlpA and AlpB have yet been identified. The *alpA/B l*ocus has also been shown to influence host cell signaling and cytokine production upon infection. Deletion of *alpA/B *genes reduced IL-8 induction during infection with East Asian but not with Western *H. pylori *strains [34]. The *alpA/B *mutants poorly colonized the stomachs of C57BL/6 mice and were associated with lower mucosal levels of induced KC (the mouse name for human IL-8) and IL-6 [34]. In contrast to these results, in another recent study *alpA *and *alpB *gene mutants of *H. pylori *SS1 induced more severe inflammation than the parental strain in infected gerbils [33].

## OipA

OipA (outer inflammatory protein A), encoded by the *hopH *gene, was initially identified as a surface protein that promoted IL-8 production in a T4SS-independent fashion [35]. OipA expression by *H. pylori *was shown to be significantly associated with the presence of duodenal ulcers and gastric cancer, high *H. pylori *density, and severe neutrophil infiltration [36]. Later studies identified that *hopH *knockout mutant strains adhered significantly less to gastric cancer cell lines, AGS and Kato-III, than wild-type strains, and complementation of the *hopH *gene restored the adherence properties of the *hopH *mutant [37]. The presence of *oipA *has also been shown to clearly enhance production of IL-8 *in vitro *but only in the presence of the *cag*PAI [32]. Further insights came from infection studies for up to 52 weeks in the Mongolian gerbil model system. All infected gerbils developed gastritis; however, inflammation was significantly attenuated in animals infected with the Δ*cagA*, but not the single Δ*vacA *or Δ*oipA *strains [38]. However, inactivation of *oipA *decreased nuclear localization of β-catenin, a factor involved in transcriptional up-regulation of genes implicated in carcinogenesis, and reduced the incidence of cancer in the gerbils. OipA expression was detected significantly more frequently among *H. pylori *strains isolated from human subjects with gastric cancer precursor lesions versus persons with gastritis alone [38]. The host receptor for OipA, however, remains unknown.

## HopZ

The *hopZ *gene encodes a protein which was shown by immunofluoresence to be located on the surface of the bacteria. A knockout mutant strain showed significantly reduced binding to the AGS cell line, compared to the corresponding wild-type strain [39]. Lack of production of HopZ did not affect the ability of the bacterium to colonize the stomachs of guinea pigs [40]. However, a role for HopZ in colonization *in vivo *has recently been proposed as deletion of *hopZ *reduced the ability of *H. pylori *to survive in a germ-free transgenic mouse model of chronic atrophic gastritis [41]. In addition, one of the few differences identified in *H. pylori *strains isolated from infected volunteers, was an OFF/ON switch in the phase-variable *hopZ *gene suggesting strong *in vivo *selection for HopZ during colonization [42]. Similar to OipA, the host receptor for HopZ has not yet been identified and will be a major challenging aim for future research.

## Role of the *cag*PAI in cell adhesion and T4SS function

### Composition of the *H. pylori *T4SS apparatus

The T4SS in the *cag*PAI belongs to a large group of transmembrane transporters that are ancestrally related to plasmid DNA conjugation systems of Gram-negative bacteria and have been found in many pathogenic and non-pathogenic organisms [9,43,44]. Although evolutionary conserved, T4SSs are functionally heterogenous in respect to both the delivered substrate (DNA-protein complexes or proteins) and the involved recipients. Recipients can be either bacteria of the different or same species, or species from other kingdoms including plants, fungi and mammalian cells. Besides *H. pylori*, T4SSs have also been found in *Agrobacterium*, *Legionella*, *Bartonella, Bordetella *and other pathogens, and typically consist of a distinct set of VirB/D proteins. The latter include the VirB1-VirB11 components and the so-called coupling factor, the NTPase VirD4. The agrobacterial T-DNA system is the prototype of a T4SS transporter and its VirB proteins have been classified into three groups: (i) the putative core or channel subunits (VirB6-10), (ii) the energetic components (the NTPases VirB4 and VirB11) and (iii) the pilus-associated proteins (VirB2, and possibly VirB3 and VirB5). VirB1 is a proposed transglycosylase for limited lysis of the murein layer at the T4SS assembly site in the membrane [45,46]. In case of the *H. pylori *T4SS, all orthologs of the 11 VirB proteins and VirD4 as well as some accessory factors have been identified to be encoded by the *cag*PAI [10,47,48]. Mutagenesis of all individual *cag*PAI genes revealed at least 14 essential and two accessory components while some other genes are not required for injecting CagA [9,49,50]. The function of many accessory T4SS factors is not yet clear, however, the role of CagF and CagD was recently elucidated. CagD appears to serve as a potential multifunctional component of the T4SS which may be involved in CagA injection at the inner membrane and may localize outside the bacteria to promote other responses in host cells [51]. In addition, CagF is a chaperone-like protein for CagA that binds close to the carboxy-terminal secretion motif of the effector protein, which is important for its translocation by the T4SS [52,53]. Further studies using yeast two-hybrid technology, fractionation and immunoprecipitation approaches identified selective interactions of numerous *cag*PAI proteins which are likely to have an important role in early T4SS assembly steps [50,54].

### Crystal structure of the T4SS core complex and several *cag*PAI proteins

A major contribution to our current knowledge about T4SS nanomachineries in bacteria came from resolution of crystal structures of the T4SS-core from plasmid pKM101 [45,55]. Three proteins (VirB7, VirB9 and VirB10) assemble into a ~1 megadalton structure spanning the inner and outer membranes. This core structure consists of 14 copies of each of the proteins and forms two layers, inserting in the inner and outer membrane, respectively [45]. The crystal structure of a ~0.6 megadalton outer-membrane complex containing the entire O layer was solved at higher resolution [55]. Comparison of the structures points to conformational changes regulating T4SS channel opening and closing, which could be involved in the transport of effector molecules [45,55]. In addition to these major findings, the crystal structures of four individual structural *cag*PAI proteins have been reported. The structure of VirB11 revealed a hexameric ring complexed with the regulatory protein HP1451, which functions as a gating factor in the inner membrane, proposed to cycle through closed and open conformations as triggered by ATP-binding and ATP-hydrolysis, respectively [56-58]. The crystal structures of CagS, a 23-kDa protein coded by a well-conserved *cag*PAI gene whose exact function remains elusive, and CagZ, a 23-kDa protein involved in the translocation of CagA, have also been solved [59,60]. Moreover, the structural characterization of CagD indicated that it exists as a covalent dimer in which each monomer folds as a single domain that is composed of three α-helices and five β-strands [51]. In addition, the structure of CagL has been modelled based on the crystal structure available from TraC of pKM101, another VirB5 ortholog [47]. CagL seems to form a three α-helical bundle structure with an exposed domain, which is in agreement with its published circular dichroism (CD) spectrum that revealed ~65% helical sequences [61].

Finally, the 2.2-Å crystal structure of a carboxy-terminal part of CagA in complex with one of its cellular targets, the human kinase Par1b/MARK2, was recently solved [62]. The CagA peptide interacted with the kinase as an extended coil. The visible 14-amino acid peptide sequence spanned the "FPLKRHDKVDDLSK" motif, a sequence occurring twice in the crystallized CagA construct. This CagA peptide was named MKI (for MARK2 kinase inhibitor) in analogy to PKI, a well-described peptide inhibitor of protein kinase A. Interestingly, the manner in which the MKI sequence of CagA binds in the substrate-binding cleft of Par1b/MARK2 is reminiscent of the manner by which PKI binds to and inhibits PKA. Taken together, the first CagA substructure revealed that it mimics host cell kinase substrates, using a short MKI peptide to attach to the substrate binding site of Par1b/MARK2 [62]. However, injected CagA also interacts with many other host cell proteins, involved in multiple signalling cascades (Figure 1A), which are discussed elsewhere [7,8].

### Structure of the T4SS apparatus in live *H. pylori*

Electron microcopy of infecting *H. pylori *has demonstrated that assembly of the T4SS is induced after host cell contact and represents a needle-like structure extending from the bacterial outer membrane, also called T4SS-pili [61,63,64]. These pili are proposed to consist of CagC, which was identified as the major VirB2 pilin subunit ortholog [65], however, direct labeling of the pili with α-VirB2 antibodies was not yet demonstrated. Studies using antibody-labelling with immunogold have shown that the bacterial protrusions are decorated by VirB10 (CagY) [64] and VirB5 (CagL) [61]. CagY proteins are about 250-kDa in size and can differ enormously in size between strains and changes size during colonization of a given host. In-frame deletions or duplications rearrangements in VirB10 can result in reduced host antibody recognition which may allow immune evasion [66]. The T4SS-needle base can be stained with antibodies against VirB7 (CagT) and VirB9 (CagW) proteins [63,64]. In addition, immunogold-staining indicated the presence of CagA at the tip of the appendages, providing the first direct evidence that CagA may be indeed delivered through the pilus, an observation not yet reported for T4SS substrates in other bacteria [61]. However, transport of CagA through the T4SS is proposed to occur by an energy-dependent mechanism stimulated by the concerted action of NTPases VirB4, VirB11 and VirD4 [46,56,67].

There are two T4SS-pilus assembly models proposed for *H. pylori*. As outlined above, all orthologs of the 11 VirB proteins and VirD4 have been identified to be encoded in the *cag*PAI as well as some accessory proteins [10], leading to a T4SS model similar to that of the agrobacterial T4SS (Figure 1B, left). In line with these conclusions, immunogold-labelling studies indicated that the tips of the T4SS pilus are decorated with CagL/VirB5 [61], which exhibited a similar distribution of VirB5 orthologs on the T4SS pilus in *Agrobacterium *[68]. In a second model (Figure 1B, right), it has been proposed that the appendages in *H. pylori *are covered locally or completely by CagY [64] and the T4SS includes all VirB components, except VirB5 [50]. Remarkably, CagY is a very large protein containing two transmembrane segments with the mid region (also called the repeat domain) exposed to the extracellular side like a hairpin-like structure [64]. As described above, VirB10 forms the inner core in a common T4SS model [45,55], but *H. pylori *CagY/VirB10 clearly differs from their counterparts in other T4SSs [69]. Thus, further studies are necessary to investigate if the T4SS pilus in *H. pylori *is more similar to that in *Agrobacterium *(mainly composed of VirB2 and VirB5 subunits) or if it is made-up of CagY as major pilus protein, or if it is a mix of both (Figure 1B).

### The function of the T4SS depends on the used cell system

Although *H. pylori *is a stomach-specific pathogen in humans, infection studies *in vitro *have shown that CagA can be injected into many different cell types. A summary of human cell types with reported susceptibility for the uptake of T4SS-delivered CagA *in vitro *is shown in Table 2. The major criterium for successful translocation in a given cell line is that CagA undergoes tyrosine phosphorylation (CagA^PY^) by host kinases of the Src and Abl family [70-73], which is commonly monitored in cell lysates or immunoprecipiates (IPs) using monoclonal phosphotyrosine-specific antibodies (Table 2). Interestingly, various studies noted significant cell type-specific differences in CagA^PY ^levels during infection of human cell lines. In addition, injected CagA^PY ^was reported for some cell types from mice and monkeys (Table 3), while selected other cell lines from humans, hamster or dog appeared to be resistant for detection of CagA^PY ^(Table 4). As controls, *in vitro *phosphorylation experiments of CagA with various cell lysates indicated that the kinases are active and strongly phosphorylated CagA [74]. Thus, the variation in CagA^PY ^levels during infection evidently resulted from different levels of CagA translocation [74,75]. There are several scenarios that may explain the observed host cell specificity. One potential explanation is that specific host cell factors might be required to activate the T4SS. This activation could operate at the level of protein expression. For example, this is the case for the type-III secretion apparatus in *Yersinia *species [76]. However, CagA is one of the most abundant proteins in the proteome of *H. pylori *even in the absence of host cell contact [77] indicating that the translocation process is repressed, rather than a CagA expression effect. Indeed, despite its abundant presence, CagA is not secreted into the supernatant [78]. This represents a clever resource-saving strategy reminiscent to *Shigella *species, where effector proteins are stored in the bacterial cytoplasm before contacting host cells. In the latter case, translocation is triggered by a variety of factors, such as extracellular matrix proteins, bile salts or Congo red [79]. It was therefore proposed that the *H. pylori *T4SS might be similarly activated by specific factors exposed on the surface of specific target cells [61].

**Table 2:** Reported phosphorylation/injection of CagA in human cell lines^a^

Cell line	Origin	*H. pylori *strains used	**Phospho-CagA**^**b**^	Applied methods	References
AGS	stomach	G27, P1, P12, P210, GU301, GU303, GU304, GU305, GU306, GC401, GC402, 342, ATCC43579, 87A300, NCTC11916, NCTC11637	+++	IP, Anti-PY WB, CF, IF, 2-DE, MS	[78,92-95]

MKN-1	stomach	P12	+	IP, Anti-PY WB	[75]

MKN-7	stomach	P12	+	IP, Anti-PY WB	[75]

MKN-28	stomach	P1, P12	++	Anti-PY WB/IPA	[74]

MKN-45	stomach	P1, P12	+++	Anti-PY WB/IPA	[74]

MKN-74	stomach	P12	+	IP, Anti-PY WB	[75]

KATO-3	stomach	P1, P12	+++	Anti-PY WB/IPA	[74]

23123/87	stomach	P12	++	IP, Anti-PY WB	[75]

Hs-746T	stomach	P12	++	IP, Anti-PY WB	[75]

FU-97	stomach	P12	+	IP, Anti-PY WB	[75]

NUGC-4	stomach	P12	+	IP, Anti-PY WB	[75]

SNU-1	stomach	P12	+	IP, Anti-PY WB	[75]

SNU-5	stomach	P12	++	IP, Anti-PY WB	[75]

SNU-16	stomach	P12	+	IP, Anti-PY WB	[75]

HeLa	cervix	P1, P12	++	Anti-PY WB/IPA	[74,84]

HT-29	colon	P1, P12	++	Anti-PY WB/IPA	[74]

Hec1.b	endometrium	P1, P12	+	Anti-PY WB/IPA	[74]

293T	kidney	P1, P12	++	Anti-PY WB/IPA	[74]

HL	lung	P1, P12	++	Anti-PY WB/IPA	[74]

HepG2	liver	P1, P12	++	Anti-PY WB/IPA	[74]

MCF-7	breast	P12	+++	Anti-PY WB	[72]

THP1	blood	P1, P12, ATCC43526	+++	Anti-PY WB	[96]

U937	blood	P1, P12, G27	+++	Anti-PY WB	[97]

Josk-M	blood	P1, P12, G27	+++	Anti-PY WB	[97]

B cells, primary	blood	HM-6, HM-9	+++	IP, Anti-PY WB	[98]

BJAB	blood	HM-6, HM-9	+++	IP, Anti-PY WB	[98]

^a ^Abbreviations: 2-DE (two-dimensional protein gel electrophoresis); anti-PY WB (Westernblotting of infected cell lysates using several pan-phospho-tyrosine antibodies); CF (cell fractionation); IF (localization by immunofluorescence); IP (immunoprecipitation); IPA (*in vitro *phosphorylation assays of CagA with cell lysates to ensure that active kinases are present in the cells of interest); MS (mass spectrometry of precipitated phospho-CagA).

^b ^Intensity of CagA's tyrosine phosphorylation signals: +++ strong phosphorylation; ++ moderate phosphorylation; + weak phosphorylation.

**Table 3:** Reported phosphorylation/injection of CagA in cell lines other than human ^a^

Species	Cell line	Origin	*H. pylori *strains used	**Phospho-CagA**^**b**^	Applied methods	References
Mouse	J774.A1	blood	P1, P12, G27, ATCC43526	+++	IP, 2-DE, Anti-PY WB, MS	[96,97]

Mouse	Fibroblast	embryo	P12	+	Anti-PY WB, CF	[61]

Mouse	SR 4987	bone marrow	P1, P12	+	Anti-PY WB/IPA	[74]

Mouse	L929	fibroblasts	P1, P12	+	Anti-PY WB/IPA	[74]

Monkey	Cos1	kidney	P1, P12	+	Anti-PY WB/IPA	[74]

^a ^Abbreviations: 2-DE (two-dimensional protein gel electrophoresis); anti-PY WB (Westernblotting of infected cell lysates using several pan-phospho-tyrosine antibodies); IPA (*in vitro *phosphorylation assays of CagA with cell lysates to ensure that active kinases are present in the cells of interest); IF (localization by immunofluorescence), IP (immunoprecipitation); CF (cell fractionation); MS (mass spectrometry of precipitated CagA^PY^).

^b ^Intensity of CagA's tyrosine phosphorylation signals: +++ strong phosphorylation; ++ moderate phosphorylation; + weak phosphorylation.

**Table 4:** Cell lines with reported resistance for phosphorylation/injection of CagA ^a^

Cell line	Origin	Species	*H. pylori *strains used	Applied methods	References
Hek293	kidney	human	P1, P12, 26695, P310	Anti-PY WB/SI	[99]

GLC4	Lung	human	P1, P12	Anti-PY WB/IPA	[74]

CHOK1	Ovary	hamster	P1, P12	Anti-PY WB/IPA	[74]

MDCK	Kidney	dog	P1, P12	Anti-PY WB/IPA	[74]

^a ^Abbreviations: anti-PY WB (Westernblotting of infected cell lysates using several pan-phospho-tyrosine antibodies); IPA (*in vitro *phosphorylation assays of CagA with cell lysates to ensure that active kinases are present in the cells of interest); SI (synchronised infection assays: bacteria were centrifuged onto Hek293 cells to ensure proper contact of bacteria with these host cells).

## The T4SS receptor hypothesis: role of host cell integrin

Despite the above reports, it was assumed for many years that CagA can be randomly injected into gastric epithelial cells. However, this is obviously not the case because more recent studies showed that numerous host cell surface molecules are required for T4SS function, suggesting a sophisticated control mechanism through which *H. pylori *injects CagA [80]. The first identified host receptor for the T4SS was integrin β1 [61]. Based on a series of experiments including the use of integrin β1 knockout cell lines (GD25 and GD25β1), gene silencing RNAs, function-blocking antibodies and competition experiments with a well-known integrin β1 bacterial adhesin (InvA from *Yersinia*), compelling evidence was provided that integrin β1 plays a crucial role for injection of CagA during infection of several non-polarised AGS and mouse cell lines [61]. In line with these observations, various structural T4SS proteins have been demonstrated to bind to integrin β_1 _*in vitro*, including CagL, CagA, CagI and CagY (Table 1). However, while very little is known about interactions of CagA and CagI with integrin, CagL has been investigated intensively. Like the human extracellular matrix protein fibronectin, CagL carries a RGD-motif shown to be important for interaction with integrin β_1 _on host cells, as well as downstream signaling to activate tyrosine kinases including EGFR, FAK and Src [81]. However, mutation of the RGD-motif in CagL revealed no reduction of injected CagA^PY ^during infection in another study [82]. Another unsolved question is the structure of CagY with respect to which domain is exposed to the extracellular space. While the repeat domain in the middle of CagY on bacteria has been shown to be accessible to recognition by antibodies [64], *in vitro *binding studies and yeast-two hybrid screens revealed that the very carboxy-terminus interacted with integrin β_1 _[82]. However, although it seems clear that each of the above factors exhibits an important functional role for injecting CagA, their interaction capabilities with integrin β1 during infection are unknown, and need to be investigated in future studies.

## Role of phosphatidylserine and cholesterol for injection of CagA

Another factor interacting with T4SS functions emerged from a recent study indicating that CagA binds directly to phosphatidylserine (PS) of the host cell and that this interaction is involved in CagA delivery into AGS cells based on saponin-fractionation experiments [83]. *In vitro*, the full-length protein binds to PS and this required a K-Xn-R-X-R motif present in the central region of CagA [83]. During *H. pylori *infection, the PS-binding protein annexinV and anti-PS antibodies both reduced CagA injection levels [83], suggesting that bacterial contact with PS is important for CagA delivery across the host membrane. Mutagenesis experiments showed that two arginine residues (R619 and R621) in the above motif are crucial for binding of purified CagA to PS and ensure membrane localization of transfected CagA in polarized MDCK cells [83]. Murata-Kamiya and co-workers proposed that the reported CagL-β1-integrin interaction may stabilize the CagA-PS interaction and may contribute to internalization of PS-bound CagA into host cells through activation of integrin signaling [83]. However, the injection mechanism of CagA into polarized MDCK cells is not completely clear because many other studies performed CagA transfection or biochemical fractionation experiments, but in most cases did not investigate phosphorylation of CagA [83-87]. The situation becomes even more puzzling because another group reported that MDCK cells are resistant to injection/phosphorylation of CagA upon infection [74]. Thus, the exact mechanism of CagA injection into polarized host cells requires further elucidation. Nevertheless, these findings point to the lipid bilayer in the host cell membrane as a second platform for T4SS-host cell interplay, rather then a "piercing"-like injection mechanism by the T4SS-pilus. Interestingly, recent findings from other groups indicated that cholesterol in lipid rafts, another component of the lipid bilayer, is also required for CagA translocation and pro-inflammatory signaling [88,89]. Taken together, these studies indicate that there are at least three host cell factors being involved in proper T4SS functions of *H. pylori*.

## Concluding remarks

*H. pylori *is one of the most successful human pathogens. Studies of host-bacterial interactions using their fundamental adhesins and the virulence factors CagA and T4SS have provided us with many detailed insights in processes ultimately connected to *H. pylori *colonisation and pathogenesis. The current opinion implies a model in which the major adhesins BabA, SabA and others make the initial and sustained host cell contact important for bacterial colonisation. Once intimate contact is established, the T4SS further interacts with specific host cell surface molecules including integrins and PS to facilitate injection of CagA, probably in cholesterol-rich microdomains on host cells, the lipid rafts. The above discussed studies indicate that at least three known host factors are involved in CagA injection (integrin, PS and cholesterol). However, it also seems clear that some cell lines are resistant to injection of CagA indicating that none of the reported host factors alone can fascililtate the injection process. We therefore assume that the T4SS injection mechanism is much more complicated than originally proposed and probably requires even more host factors, probably acting cooperatively. It should also be noted that almost all of the functional T4SS studies have been made *in vitro *using cultured cell lines, which are indeed very helpful. However, we are aware of only one *in vivo *study, in which phosphorylated CagA was isolated from biopsies of atrophic gastritis and in noncancerous tissues from *H. pylori*-positive patients using immunoprecipitation and Western blotting approaches [90]. Thus, more studies are clearly necessary to investigate under which circumstances and how CagA is injected during infection *in vivo*. In particular, it remains to be investigated if CagA injection occurs at the apical, basolateral and/or other sites of the gastric epithelium. In addition, it has been convincingly shown that CagA can be very efficiently translocated into certain immune cells *in vitro *(Table 2). Thus, future studies are necessary to investigate the importance of these findings *in vivo*. Finally, the evolutionary advantage of the T4SS for *H. pylori *is also not yet clear and needs to be investigated more thoroughly. For example, recent studies indicated that injected CagA enables *H. pylori *to grow as microcolonies adhered to the host cell surface even in conditions that do not support growth of free-swimming bacteria [91]. Thus, it appears that the *H. pylori *T4SS will continue to be a fascinating and rewarding research topic in future studies.

## Competing statement

The authors declare that they have no competing interests.

## Authors' contributions

SB, MC and NT all contributed to the writing of this manuscript. All authors read and approved the final version of this manuscript.
